# Infusing zeta inhibitory peptide into the perirhinal cortex of rats abolishes long-term object recognition memory without affecting novel object location recognition

**DOI:** 10.3389/fnbeh.2022.1007748

**Published:** 2022-12-06

**Authors:** Keanan Augereau, Paola V. Migues, Oliver Hardt

**Affiliations:** Department of Psychology, McGill University, Montréal, QC, Canada

**Keywords:** perirhinal cortex, object recognition, memory maintenance, long-term memory, PKMzeta inhibitors

## Abstract

Infusing the amnesic agent zeta inhibitory peptide (ZIP) into the dorsal hippocampus disrupts established long-term object location recognition memory without affecting object identity recognition, which likely depends on the perirhinal cortex. Here, we tested whether infusing ZIP into the perirhinal cortex can abolish long-term memory supporting object identity recognition, leaving long-term object location recognition memory intact. We infused ZIP into the perirhinal cortex of rats either 1 day or 6 days after exposing them to two identical objects in an open field arena. One day after ZIP infusion, that is, 2 or 7 days after object exposure, we either assessed whether the animals recognized that now one of the two objects was novel or whether they recognized that one of the two familiar objects was at a new location. Our results show for both retention intervals, infusions of ZIP into the perirhinal cortex impaired novel object recognition but spared novel object location recognition. Rats that received a scrambled version of ZIP had no deficit in either test at both retention intervals and expressed stronger novel object recognition compared to rats infused with ZIP. These findings support the view that object recognition depends on dissociable memory representations distributed across different brain areas, with perirhinal cortex maintaining long-term memory for what objects had been encountered, and hippocampus supporting memory for where these objects had been placed.

## Introduction

The question of which brain structures are essential for object recognition memory has been controversially debated because results from humans, non-human primates, and rodents have not always been in agreement (Brown and Banks, 2014; Cohen and Stackman, 2015). The overwhelming evidence seems to identify the perirhinal cortex as the area required for hosting long-term memory representations supporting object recognition (Winters and Bussey, 2005; Winters et al., 2008). It is less clear whether the hippocampus also supports object recognition memory, as some studies indicate its involvement in object recognition tasks, and some studies suggesting otherwise (Warburton and Brown, 2015). While these variations sometimes depend on specific task conditions, they may also arise from differences in the methods used to interfere with processes in the hippocampus. For example, lesions and pharmacological interventions have led to different conclusions regarding the role of the hippocampus in object recognition memory (Broadbent et al., 2004; Brown et al., 2010; Cohen and Stackman, 2015).

This complex situation may benefit from an approach that targets established memory, after memory formation and consolidation have concluded, outside the context of other memory processing phases such as retrieval and reactivation. This can be achieved with the amnesic agent zeta inhibitory peptide (ZIP), which has been designed to transiently block the activity of protein kinase M zeta (PKMζ), an autonomously active protein kinase C (PKC) isoform. Several lines of evidence suggest that PKMζ contributes fundamentally to various forms long-term memory. For example, infusing ZIP into the hippocampus can impair consolidated spatial memories (Pastalkova et al., 2006; Serrano et al., 2008; Migues et al., 2010), and infusing it into the basolateral nucleus of the amygdala can disrupt long-term auditory (Migues et al., 2010) as well as contextual fear memory (Serrano et al., 2008). We have previously shown that infusing ZIP into the dorsal hippocampus disrupts long-term memory supporting the recognition of novel object locations, without affecting the ability to recognize novel objects (Hardt et al., 2010), which likely depends on perirhinal cortex.

To complement these results, here we tested whether infusing ZIP into the perirhinal cortex—at time points when it cannot affect memory formation or retrieval—impairs novel object recognition while sparing novel object location recognition.

## Methods

### Animals

We obtained male Long-Evans rats at 250–300 g from Charles River, Canada, and housed them in pairs with environmental enrichment (wooden gnawing block, PVC tube) in transparent plastic cages. Rats consumed food and water *ad libitum*. The light in the animal colony went on at 7 A.M. and off at 7 P.M. We performed our experiments between 9 A.M. and 2 P.M. All procedures followed the relevant guidelines published by the Canadian Council on Animal Care, and the Faculty Animal Care Committee at McGill University reviewed and approved them.

### Surgery

Surgeries followed the procedures used in our earlier experiment testing the role of PKMζ in object location memory in the dorsal hippocampus (Hardt et al., 2009, 2010). We gave rats intraperitoneal injections of an anesthetic mixture consisting of xylazine (3.33 mg/ml), ketamine (55.55 mg/ml), and Domitor (0.27 mg/ml) in a volume of 1 ml/kg. Once animals were in deep anesthesia, we shaved their heads and then placed them into a stereotactic frame (David Kopf Instruments, Tujunga, CA, USA). A midline incision exposed the skull, and we implanted three jeweler screws and two guide cannulas (22 gauge, P1 Technologies, Roanoke, VA, USA) into each hemisphere aiming at the perirhinal cortex (pRh) at coordinates A/P -5.5 mm, M/L 6.6 mm, D/V -6.7 mm (Paxinos and Watson, 2004). We applied dental cement to stabilize the cannulas and inserted obturators to prevent blocking and contamination. Thirty minutes before the end of surgery, we injected subcutaneously the analgesic Carprofen. We reversed anesthesia with an intraperitoneal injection of Antisedan (7.5 mg/kg). We let rats recover from surgery for 7 days, during which we handled them and regularly cleaned the obturators with 70% ethanol in sterile water.

### Drug infusions

Zeta inhibitory peptide (Myr-SIYRRGARRWRKL-OH, Anaspec) or scrambled ZIP (scrZIP); (Myr-RLYRKRIWRSAGR-OH; Anaspec) were dissolved in 100 mM Tris-saline to a final concentration of 10 mM and the pH was adjusted to 7.2. We infused 1 μl (10 nmol) of the peptide solution bilaterally into the perirhinal cortex using 28-gauge microinjectors (P1 Technologies, Roanoke, VA, USA), connected with polyethylene tubing to a Hamilton syringe, at a speed of 0.25 μl/min (i.e., a total volume of 1 μl in 4 min). After the infusion, we left the microinjectors in place for 90 s to allow drugs to diffuse away from the injector tip. Between animals, we cleaned the microinjectors with 70% ethanol in sterile water and thoroughly dried them with paper towels.

### Apparatus

We used an open field measuring 60 × 60 × 60 cm, made of laminated particle board, placed onto a wooden platform 10 cm above the floor. A digital camera positioned 130 cm above the field recorded each trial. The open field was in the center of a room measuring 3 x 3 m, having no windows and one red door. There were no other salient distal cues in the room. The indirect lighting produced 15 lux, measured at the floor of the open field. The floor of the open field was covered with about 4 cm of the same type of sawdust bedding also used for the home cages. We changed the bedding in the open field between experiments. As stimuli, we used several everyday (junk) objects with no known biological significance for the rats. The objects were glued to the bottom of mason jars, and the top of the jars was then secured by screwing the jars into jar lids, which were fastened to the floor with screws and wing nuts (Hardt et al., 2010; Migues et al., 2010, 2014). To keep track of each copy of an object, we wrote a number onto the rim of the mason jar to which it was glued, so that rats were not able to see these identifying marks.

### Behavioral procedures

We ran two studies consisting of two experiments each; one study tested memory after a 2 days memory retention interval, the other one tested memory after a 7 days interval. For each study, we used a new group of rats. Each study had two experiments and the rats used for a study took part in both. In each study, one experiment tested for object identity recognition memory, the other one for object location recognition memory. There were between 10 and 14 days between each experiment, and their order (i.e., whether we assessed novel object or novel location recognition first) was counterbalanced. We neither used objects nor locations twice for any given rat in the two studies, and we used a different open field in a different room for each study.

All experiments had four phases—Habituation, Sampling (i.e., training), Drug Infusion, and Probe (i.e., memory test). The procedures used in the experiments closely followed those we used in our earlier study (Hardt et al., 2010).

#### Habituation

About 7 days after surgery, we habituated the rats to the open field over four consecutive days. Each day, we placed rats for 10 min into the open field. The open field contained two identical copies of the same object during all 4 days of Habituation, but the position of the two objects changed from day to day. We put objects always in opposing corners (i.e., NE-SW or NW-SE). We lowered rats into the open field with their head facing an empty corner. Between rats, we removed the objects, cleaned them with 70% ethanol in distilled water, removed feces from the arena, and swirled the arena floor bedding around to disperse any possible odor markings left behind.

#### Sampling

One day after the last Habituation trial, we presented animals with two copies of an object that they had not seen before. We placed the objects into opposing corners and they stayed at the same position throughout all Sampling trials. For Exps 1 and 2, we trained rats for two consecutive days, twice each day, with one session in the A.M., another one about 4–5 h later in the P.M. For Exps 3 and 4, we trained them for seven consecutive days, 10 min per day, during the A.M. phase of the day. Sampling trials were always 10 min long. We lowered rats into the open field, as during Habituation, facing a corner that had no object.

#### Infusions

For Exps 1 and 2, rats received one infusion the day after the last sampling session. For Exps 3 and 4, rats received the infusion 6 days after the last sampling session. Infusions occurred between 11 A.M. and 1 P.M. in the home colony of the animals.

#### Probe

One day after the infusion, i.e., 2 days after Sampling in the first study (Exps 1 and 2), and 7 days after Sampling in the second study (Exps 3 and 4), we tested long-term recognition memory. To assess object identity recognition (Exps 1 and 3), we presented rats with another copy of the object used during Sampling and one novel object, both placed where objects had been before. Novel and familiar objects were counterbalanced across conditions. To assess location novelty recognition (Exps 2 and 4), we presented rats with the same objects used during Sampling but moved one of them to a novel location. In each Probe trial, we lowered rats into the open field facing a corner that did not contain an object, and that was furthest away from both objects (the latter only relevant for object location recognition tests). Each Probe trial took 3 min.

### Histology

**FIGURE 1:**
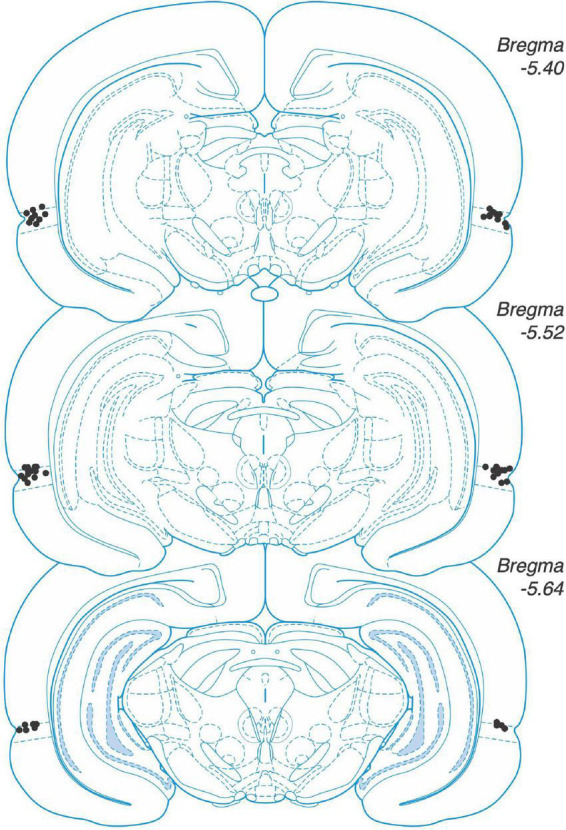
Placement of microinjector. tips in perirhinal cortex. Placements shown on coronal sections from rat brain atlas by Paxinos and Watson (2004).

We deeply anaesthetized animals and then decapitated them. We removed the brains and fixed them in a mixture of 4% paraformaldehyde and 30% sucrose–saline. We used a cryostat to obtain sections of 50 μm thickness. We verified the placement of the implanted cannulas with a light microscope. We included animals in the analyses when an experimenter blind to the treatment group detected the injector tips inside perirhinal cortex in both hemispheres (Figure 1).

### Data analysis

We manually scored the recorded videos. We considered rats exploring objects when they directed their nose at an object at an angle of at least 45 degrees and no farther away than 2 cm. We did not consider sitting and climbing onto objects as exploratory behavior (Ennaceur and Delacour, 1988). We scored the videos and then pre-processed the data with in-house software prior to statistical analyses. To determine novelty preference (*d*), we measured the total amount of time rats spent exploring the novelty (*t_new*; object or location) and the familiarity (*t_old*), and calculated *d* as *d* = (*t_new* – *t_old*)/(*t_new* + *t_old*). The novelty preference ratio *d* can take any value between -1.0 and 1.0, with *d* = 0 denoting equal exploration of both novelty and familiarity, i.e., the absence of exploratory preferences and suggesting that animals do not express memory for the object locations or object identity, respectively, from the training phase; values of *d* significantly higher than 0 indicate that rats express memory. We used Jamovi (version 2.3)^1^ for our statistical analyses. All data were normally distributed, and therefore we used *t*-tests or repeated-measures ANOVAs to determine significant group differences and one-sample *t*-tests to compare *d* against 0 to determine whether groups expressed memory. The threshold for accepting the null hypothesis was set to alpha = 0.05. For significant effects, we report the effect size measures partial eta squared (*η^2^_p_*), or Cohen’s d.

## Results

### Infusing zeta inhibitory peptide into the perirhinal cortex impairs recent (1 d old) long-term object but not object location recognition memory

**FIGURE 2:**
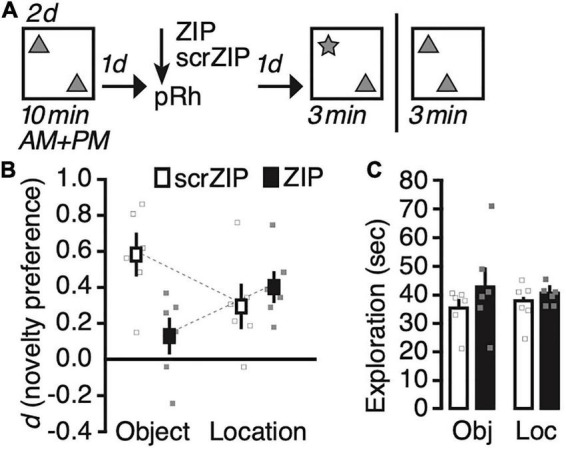
Infusing zeta inhibitory peptide (ZIP) into the perirhinal cortex impairs recent (1 day old) long-term object recognition memory but not object location recognition memory. **(A)** Experimental protocol. We trained rats twice per day on two consecutive days, exposing them for 10 min to two copies of an object in an open field arena. One day later, we infused ZIP (10 nmol) or scrZIP (10 nmol) into the perirhinal cortex. The following day, we put animals back into the open field. For half of the rats, we replaced one familiar object with a novel one (assessing novel object recognition), for the other half, we moved one object to a different location (assessing novel object location recognition). Rats took part in both assessments, with about 10–14 days between experiments (i.e., between memory test and habituation). **(B)** Infusing ZIP, but not scrambled ZIP (scrZIP) into the perirhinal cortex impairs the expression of object recognition memory, but not object location memory. **(C)** Exploratory activity during the test was the same for both groups in each assessment. Error bars ± 1 standard error of the mean.

We exposed rats for 2 days to two identical copies of an object in our test arena (Figure 2A), and infused ZIP or the scrambled version scrZIP into the perirhinal cortex 24 h later. One day after the infusion into perirhinal cortex, we tested half the rats of each drug infusion group for their memory of what object had been presented (object identity) or memory for where the objects had been placed (location memory). Between 10 and 14 days after the memory test, we repeated the experiment, infusing the same drug into the same rats, testing them for the memory type we had not assessed before (i.e., location or identity, respectively). A repeated-measures ANOVA on novelty preference (*d)* with memory type (identity vs. location) as the repeated factor and treatment (scrZIP vs. ZIP) as between-subjects factor revealed a significant interaction, *F*(1,10) = 7.0, *p* = 0.02, *η^2^_p_* = 0.41, but no significant main effect of memory type, *F* < 1, or treatment, *F*(1,10) = 3.7, *p* = 0.08. *Post-hoc* tests (Tukey) determined that rats infused with ZIP expressed a significantly lower preference to explore the novel object compared to rats that received scrZIP, *t* = –3.2, *p* < 0.04; no other comparison was significant. One sample *t*-tests comparing novelty discrimination against what would be expected by chance alone (i.e., zero) determined that that rats infused with ZIP, *t*(5) = 5.0, *p* = 0.004, Cohen’s d = 2.0, and rats infused with scrZIP, *t*(5) = 2.7, *p* = 0.04, Cohen’s d = 1.1, preferred to explore the object at the novel location; however, only rats infused with scrZIP also significantly preferred to explore the novel object, *t*(5) = 5.5, *p* = 0.003, Cohen’s d = 2.3 [ZIP group: *t*(5) = 1.4, *p* = 0.23]. A repeated-measures ANOVA on exploratory activity with memory type (identity vs. location) as the repeated factor and treatment (ZIP vs. scrZIP) as between-subjects factor revealed no significant effects (memory type: *F* < 1; treatment: *F*(1,10) = 2.4, *p* = 0.15; interaction: *F* < 1). Taken together, these results suggest that infusing ZIP into the perirhinal cortex can impair 2 days old recognition memory for “what” objects had been encountered, leaving intact long-term recognition memory for “where” objects had been placed. The absence of differences in exploratory activity between the groups in each memory test indicates that differences in motivation or motility cannot account for the pattern of novelty preference during the tests.

### Infusing zeta inhibitory peptide into the perirhinal cortex impairs remote (7 days old) long-term object but not object location recognition memory

**FIGURE 3:**
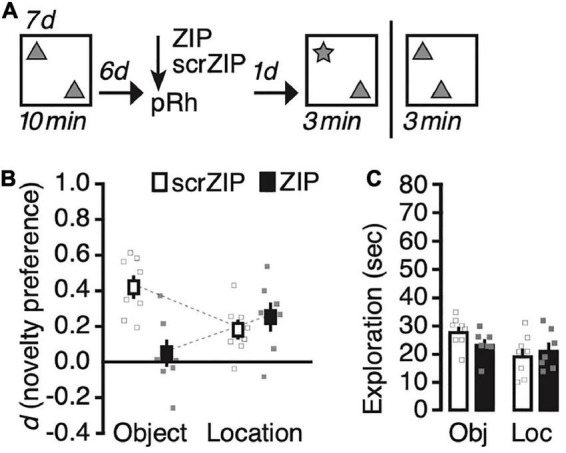
Infusing zeta inhibitory peptide (ZIP) into the perirhinal cortex impairs remote (6 days old) long-term object recognition memory but not object location recognition memory. **(A)** Experimental protocol. We trained animals for seven consecutive days, exposing them 10 min per day to two copies of the same object in an open field arena. Six days later, we infused ZIP (10 nmol) or scrZIP (10 nmol) into the perirhinal cortex. The following day, we assessed memory and repeated the experiment memory as before (Figure 2). **(B)** Infusing ZIP into the perirhinal cortex impairs novel object recognition, but not novel location recognition. **(C)** Rats explored objects more in tests in which a novel object was present than when a familiar object moved to a new location, irrespective of whether they receive ZIP or scrZIP. Error bars ± 1 standard error of the mean.

We next explored whether ZIP will impair novel object recognition for more remote long-term memories. We changed the training procedure to promote the formation of location memory that lasts for at least 7 days (Migues et al., 2016), and adjusted the retention interval accordingly (Figure 3A). During Sampling, we exposed rats to two copies of an identical object—the same we used for the first study—daily for 10 min for seven consecutive days, then infused ZIP or scrZIP into the perirhinal cortex 6 days after the last training session; finally, we tested recognition memory the following day, 7 days after the last Sampling trial. As before, we tested half the rats on object location recognition memory and half on object identity recognition memory, and repeated the experiment between 10–14 days later, as described above, then testing rats for the memory we had not assessed already. A repeated-measures ANOVA on novelty preference (*d*) with memory test (identity vs. location) as the repeated factor and treatment (scrZIP vs. ZIP) as the between-subjects factor detected a significant interaction, *F*(1,13) = 17.8, *p* = 0.001, *η^2^_p_* = 0.58, no significant main effect of test, *F* < 1, nor a significant main effect of treatment, *F*(1,13) = 4.2, *p* = 0.06, which, however, approached significance and suggested that novelty preference in general tended to be stronger in animals that received infusions of scrZIP into the perirhinal cortex, as compared to rats that received ZIP (Figure 3B). *Post-hoc* tests (Tukey) to further analyze the significant interaction revealed that rats receiving scrZIP preferred to explore the novel object significantly more so than animals that received ZIP, *t* = 4.1, *p* = 0.007. Also, novelty preference was significantly stronger for novel objects than for novel object locations in animals that received scrZIP, *t* = 3.3, *p* = 0.029. No other comparison was significant. One-sample *t*-tests comparing novelty preference against zero detected that rats infused with scrZIP and ZIP both preferred to explore the object at the novel location [scrZIP: *t*(7) = 3.6, *p* = 0.009, Cohen’s d = 1.3; ZIP: *t*(6) = 3.3, *p* = 0.016, Cohen’s d = 1.3], but that only rats infused with scrZIP also preferred to explore the novel object, *t*(7) = 7.2, *p* < 0.001, Cohen’s d = 2.6 (ZIP: *t* < 1). A repeated-measures ANOVA on exploratory activity with memory test (identity vs. location) and treatment (scrZIP vs. ZIP) as the between-subjects factor detected a significant main effect of memory test, *F*(1,13) = 6.6, *p* = 0.023, *η^2^_p_* = 0.34, no significant main effect of treatment, *F* < 1, and no significant interaction, *F*(1,13) = 1.9, *p* = 0.19 (Figure 3C). These results suggest that 6 d after acquisition, infusing ZIP into the perirhinal cortex can impair long-term memory for object identity, an intervention that does not impair the ability to recognize novel locations of objects. Our findings further imply that at this time point, situations in which rats encounter novel objects provoke more exploratory activity than situations in which a familiar object occupies a new location, irrespective of whether animals received ZIP or scrZIP into perirhinal cortex.

## Discussion

We tested whether infusing ZIP into the perirhinal cortex of rats can disrupt long-term memory that supports identification of novel objects and novel object locations in a standard object recognition paradigm. We found that this intervention impairs the expression of recognition memory for novel objects, but not novel object locations, for both recent (1 day old) as well as remote (6 days old) memories. We thus replicate the findings of Outram et al. (2022), lending strong support to our respective results. Together with our earlier demonstration that infusing ZIP into the dorsal hippocampus disrupts long-term object location but not object identity memory, these data show that long-term memory for *what* has been encountered requires representations in perirhinal cortex, while memory for *where* things had been placed requires representations in dorsal hippocampus (Winters, 2004; Forwood et al., 2005).

Although our results add to the well-established position that perirhinal cortex critically supports object recognition, it seems likely that the hippocampus is involved in object recognition memory during different memory processing phases, or memory states, as well. For example, lesions to the hippocampus impair, but not abolish, novel object recognition, in that sham-operated rats show a stronger novel object preference than rats who had received lesions to the hippocampus (Broadbent et al., 2009). Furthermore, under certain conditions, impairing dorsal hippocampal function can disrupt long-term object recognition memory. For instance, when the environment in which rats originally acquired object memory is modified when rats are briefly re-exposed to the objects they had encountered there earlier, subsequent infusions of the protein-synthesis inhibitor anisomycin into the dorsal hippocampus impair novel object recognition in a later memory test; absent changes to the context, this reactivation treatment leaves object recognition memory intact (Winters et al., 2011). Similar findings have been reported for interventions that disrupt the activity of PKMζ. For example, blocking PKMζ with ZIP or antisense in the dorsal hippocampus does not affect long-term novel object recognition memory unless these memories have been retrieved, or reactivated (Rossato et al., 2019). Recent findings in mice further suggest that the extent to which animals explore objects during the initial encounter moderates whether hippocampus or perirhinal cortex critically support long-term novel object recognition, such that longer exploration times engage the hippocampus, while shorter times recruit the perirhinal cortex (Cinalli et al., 2020). These exemplary findings suggest that in the normal brain, although the perirhinal cortex hosts memory representations necessary for novel object recognition, other brain areas, under certain conditions or during certain memory phases, such as acquisition, expression, and updating, also can critically contribute to the expression of novel object recognition or the processing of memory representations underpinning the recognition of novel objects. Thus, affecting interactions of these brain areas during certain mnemonic processing periods may result in acute or long-lasting modulation of the ability to recognize objects as being novel.

We used ZIP in our experiments because it has been widely shown to impair memory maintenance in a variety of tasks and animal models (Patel and Zamani, 2021). Several studies support the notion that ZIP disrupts long-term memory because it blocks the activity of PKMζ, promoting the internalization of GluA2-containing AMPA receptors (GluA2/AMPARs) from post-synaptic densities, thus rapidly reducing synaptic potentiation induced by learning and memory formation (Migues et al., 2010, 2014; Dong et al., 2015). It should be noted that whether PKMζ is the essential element of this maintenance processes, or whether other PKC isoforms are also recruited (Ren et al., 2013) has been controversially discussed and remains to be fully resolved (Cai et al., 2011; Kwapis and Helmstetter, 2013; Lee et al., 2013; Volk et al., 2013; Tsokas et al., 2016; Wang et al., 2016).

Irrespective of the mode of action, ZIP has the advantage that it can be administered at times when it unlikely affects other processes that could account for memory loss, such as acquisition, formation, expression, and the like, such that memory deficits can be attributed to impaired maintenance of long-term memory. There are some findings, however, that suggest that the effects of ZIP on memory retention may not arise from the assumed interaction with kinases relevant for memory maintenance, but from excitotoxic effects causing cell death (Sadeh et al., 2015), or from attenuating neural activity (LeBlancq et al., 2016). These alternative explanations could account for some of the amnesia observed with this peptide, but the results of other studies cast doubt on this interpretation. First, several studies have shown that despite ZIP-induced memory loss, animals are able to learn and form new long-term memories, suggesting that neurotoxic effects cannot readily account for the retrograde amnesia following infusions of ZIP (Pastalkova et al., 2006; Sacktor, 2008; von Kraus et al., 2010). Second, the peptide GluA2-3Y that blocks the activity-dependent removal of GluA2/AMPARs (Lee et al., 2002; Scholz et al., 2010; Migues et al., 2016) prevents the amnesic effects of ZIP, as we have shown before (Migues et al., 2016) and (Outram et al., 2022) have replicated. If indeed ZIP acts mainly via inducing excitotoxic effects, then preventing the removal of AMPA receptors from post-synaptic membranes should not block the actions of ZIP. Finally, non-specific actions of ZIP cannot account for why infusing it into the perirhinal cortex affects novel object recognition, but not the oddity discrimination task in the study of Outram et al. (2022). In conclusion, future studies could exploit this peptide to dissect the role and contributions of various brain areas during different phases of object recognition tasks more carefully (Rossato et al., 2019).

Our study thus suggests that at least some of the processes maintaining long-term object recognition memory in the perirhinal cortex involve activity of PKMζ. Notably, impairing the activity of PKMζ impairs established long-term potentiation (LTP), but not long-term depression (LTD), and it is the latter form of synaptic plasticity that has been linked to object recognition memory (Warburton et al., 2003; Griffiths et al., 2008). For example, recording from perirhinal cortex in rats, (Zhu et al., 1995) found that the second exposure to an object resulted in changed responses in a subset of the recorded neurons, such that in perirhinal cortex 13% of neurons decreased their activity, while 9% increased it. By comparison, in the hippocampus, 3% of neurons decreased their response, while 9% increased it. These data suggest that object recognition memory seems to recruit mechanisms that dampen synaptic responses in the perirhinal cortex to a larger extent than the hippocampus, linking the former to processes found in long-term depression, i.e., synaptic weakening, more so than to processes found in long-term potentiation, i.e., synaptic strengthening. Long-term depression critically depends on the internalization of GluA2/AMPARs from presynaptic membranes (McCormack et al., 2006; Diering and Huganir, 2018), and, to study its role in object recognition memory Griffiths et al. (2008) targeted this process in perirhinal cortex. Using a lentiviral vector to express a peptide in perirhinal cortex that interferes with the binding of the clathrin adaptor protein AP2 and GluA2 – an event required for GluA2/AMPAR internalization – they impaired object recognition memory in rats. Because rats acquired object recognition memory while the peptide was being expressed, the outcomes of this study cannot address whether acquiring, maintaining, or expressing object recognition memory requires GluA2/AMPAR endocytosis, yet it suggests that LTD contributes to this type of memory.

Taken together, these data suggest that processes underpinning LTD also promote object recognition memory in perirhinal cortex. Our data, as well as the findings from Outram et al. (2022), however, indicate that forms of synaptic plasticity involved in LTP also are critical for object recognition memory in this brain region. Specifically, the results of Outram et al. (2022) show that the amnesic effects of ZIP on object recognition memory involve the internalization of GluA2/AMPARs, suggesting that maintaining long-lasting object recognition memory depends on forms of synaptic plasticity that are critical for LTP, but not LTD. Thus, while these and our results seem in conflict with earlier findings, they make sense from the position that memory reflects patterns of synaptic connectivity arising from adjusting synaptic weights, i.e., the strengthening and weakening of synaptic connections, requiring processes involved in LTP as well as those involved in LTD (Norman, 2010). Future studies could address how the interplay of various forms of synaptic plasticity supports the formation and maintenance of long-term object recognition memory in the perirhinal cortex.

In summary, our findings support the view that different brain areas support memory of *what* was encountered *where* that is assessed in novelty recognition tests. Clearly, when animals explore an environment they acquire, without externally provided reinforcement, complex memories about objects and their spatial relations, with the former involving perirhinal cortex and the latter hippocampus, among other brain areas. This distributed representational nature might help explain why disrupting hippocampal processing can impair newly acquired or reactivated object recognition memory (Winters et al., 2011). Our findings lend further support for this perspective, indicating that object recognition memory represents a mnemonic capacity that relies on interactions of various brain regions, notably prefrontal cortex, hippocampus, and perirhinal cortex (Bussey et al., 2005; Murray et al., 2007; Cowell et al., 2010; Saksida and Bussey, 2010; Warburton and Brown, 2015; Chao et al., 2020). As such, it presents a well-suited rodent paradigm to study regions and processes likely underpinning human episodic and semantic memory, as others have noted before.

## Data availability statement

The raw data supporting the conclusions of this article will be made available by the authors, without undue reservation.

## Ethics statement

The animal study was reviewed and approved by Faculty Animal Care Committee of McGill University.

## Author contributions

OH and PM designed study. OH and KA conducted behavioral studies and analyzed data. All authors wrote the article and approved the submitted version.

## Footnotes

1
https://www.jamovi.org
